# The Role of Individualized Exercise Prescription in Obesity Management—Case Study

**DOI:** 10.3390/ijerph182212028

**Published:** 2021-11-16

**Authors:** Márton Dvorák, Miklós Tóth, Pongrác Ács

**Affiliations:** 1 Department of Health Sciences and Sport Medicine, University of Physical Education, 1123 Budapest, Hungary; tothmik1@hotmail.com; 2 YourPowerMed Health Center, 1015 Budapest, Hungary; 3 Institute of Physiotherapy and Sport Science, Faculty of Health Science, University of Pécs, 7621 Pécs, Hungary; pongrac.acs@etk.pte.hu; 4 Szentágothai Research Centre, University of Pécs, 7624 Pécs, Hungary

**Keywords:** adiposopathy, exercise, lifestyle medicine, body mass index, wearable technology

## Abstract

Introduction: Obesity, or adiposity-based chronic disease (ABCD), is one of the most common health risk factors nowadays. Regular exercise—part of complex lifestyle medicine program—is effective treatment for obesity but is still underestimated. Monitoring andindividualization by an exercise professional is needed to define the accurate dose effect. Materials and Methods: The 30-week lifestyle change program of a 65-year-old male patient (body mass index (BMI) 43.8 kg/m^2^) was followed by a medical doctor, exercise physiologist, and nutritionist. Over regular controls and blood tests, each training activity was measured with a heart rate monitor watch, and a diet diary was written. Results: Bodyweight decreased by 24.1 kg (18.4%) and BMI to 35.8 kg/m^2^. Decreased resting heart rate (from 72 bpm to 63 bpm), diastolic blood pressure (from 72 mmHg to 67 mmHg), and increased systolic blood pressure (from 126 mmHg to 135 mmHg) were reported, besides the reduction in antihypertensive and antidiabetic medicines. Blood test results and fitness level improved, and daily steps and time spent training increased. Conclusions: Lifestyle medicine with professional support is an effective and long-term treatment for ABCD. Individualized exercise and nutritional therapy are essential, and wearable technology with telemedicine consultation also has an important role.

## 1. Introduction

Obesity or—as it was introduced in medical literature—adiposity-based chronic disease (ABCD) or adiposopathy [1] has emerged as an important risk factor for morbidity and mortality [2,3] and reduces quality of life [4]. In the treatment of ABCD and related diseases (most commonly type 2 diabetes, cardiovascular disease (CVD), hypertension, and hyperlipidemia) lifestyle medicine intervention— principally including exercise and nutrition—must be a key element of medical care [1].

According to the WHO guidelines [5], at least 150–300 min of moderate-intensity or 75–150 min of vigorous-intensity aerobic physical activity (PA) a week is recommended for healthy adults. The prescription is the same for patients with cardiovascular and metabolic [6] diseases but also add that any PA is better than nothing. Although low-intensity PA for just 10–59 min/week also improves mortality rates [7], it is needed to spend 30 min/day engaging in moderate-vigorous exercise to have the equivalent effect to hypoglycemic and antihypertensive drugs [8,9].

Despite these recommendations, the health effects of regular exercise are still often underestimated [10]. The cause may be that the exact dose-response effect of the treatment is difficult to determine [11] because actual health status—physical condition, diseases, and medications—of the patients can be so diverse that its additional effects for health show too much variance [12]. Strength training methods have different effects for the cardiovascular system [13] and should be individualized also. Although personalized therapy that is constantly monitored and supervised by exercise professionals brings effective results in the treatment of adiposopathy and its comorbidities, research studies in this field usually work with general physical activity recommendations during interventions [14]. However, for better understanding the dose-response effect of exercise training in the case of chronic diseases, individualized prescription and fully comprehensive medical documentation are important. In this aspect, individualization means not just personalized advice at the beginning of the intervention but also longitudinal modification to match increasing physical condition and to keep motivation by changing the quantity and type of training through training modalities (i.e., methods, frequency, and intensity).

For that reason, activity and heart rate monitors are essentials in the objective measurability of exercise [15,16] with the supervision of exercise professionals and also in the maintenance of motivation during exercise therapy to reduce body weight [17]. Therefore, in this case study, all the training activities of a male patient were monitored during the 30 weeks of intervention.

The aim of this case study is to demonstrate that engaging in exercise is important and essential not only in the field of fitness but also in the field of (lifestyle) medicine. Exercise physiologists should be equal team members for healing chronic diseases as nutritionists under the supervision of medical doctors. Although medical recommendations [6] suggest lifestyle modifications as first step in case of diabetes or cardiovascular diseases, in practice, just a few patients turn to experts in nutrition and exercise.

## 2. Materials and Methods

### 2.1. Patient Introduction and Initial Conditions

In the present case study, a 65-year-old male patient with body mass index (BMI) 43.8 kg/m^2^ (obesity class III), hypertonia, prediabetes, hyperlipidemia, and knee arthrosis started a supervised and complex lifestyle medicine program. The patient was an elite football player in his twenties and, after that, exercised sparsely or not at all. Two years before the start of the program he exercised regularly: 2 × 1 h swimming and 1 h cycling a week, but it did not cause any weight loss and improvement in health. He had been smoking for 40 years but gave up five years ago. He is retired from work.

The patient had difficulty with walking due to his bodyweight and knee pain caused by arthrosis. On a few occasions a year, he experienced sudden spikes in blood pressure and dizziness for 10–15 min but tests did not explain its cause.

Previously and during the program, he has regular medical control and his medicines were appropriate for his status. At the beginning of the program, these were Astrix 100 mg, Bisoporol Sandoz 5 mg, Coverex 4 mg, Coverex-AS Komb 5 mg/1.25 mg, Fenoswiss 160 mg, and Meforal 1000 mg.

His main motivation to lose weight and “get in better shape” was the birth of his first grandchildren, which was a great factor in the success of the program.

### 2.2. Preliminary Assessments and Monitoring

The program lasted for 30 weeks. It was led by a medical doctor and supervised by a nutritionist and an exercise physiologist. Medical check-ups with blood testing were performed on weeks 0, 16, and 31. The nutritionist proposed 1600–1800 kcal and 160 g carbohydrate intake a day in the first consultation, and based on the written diet diary, she made it accurate on the further appointments on weeks 4, 12, 17, and 30. The exercise fitness test (YMCA cycling test) led by the exercise physiologist showed average resting heart rate (RHR) with 76 bpm and low fitness level (MET 5.7 mL/kg/min; VO_2_max 19.8 L/kg/min). The goal heart rate (HR) for the cardio training was defined as moderate intensity (60–70% of maximal HR) after the test.

### 2.3. Exercise Protocol

The patient participated in supervised individual training, two sessions a week. The supervised indoor training were cardio (walking, rowing, and cycling) and strength exercise (with free weight, rubber band, calisthenics exercises, and cable weight machines) for one hour/session. Before and after supervised training, blood pressure and blood sugar levels were measured. Furthermore, the patient trained at least once every day at home, which also included cycling, walking, and swimming sessions.

A smartwatch with a heart rate monitor (POLAR, Kempele, Finnland) was used during every training and home session, and data were analyzed and discussed by the exercise physiologist every week. The patient focused on just two data points on the watch during home training, which were HR during training and daily physical activity percentage, where he was asked to reach at least 100% (7 h 19 min low-intensity, 2 h 12 min medium-intensity, 58 min high-intensity physical activity, or these in combination at Level 1) every day.

## 3. Results

The results indicate positive changes in more aspects of the health and medical status of the patient. His bodyweight lowered by 24.1 kg (18.4% of the basis) and BMI decreased to 35.8 kg/m^2^. Blood test results showed improvement (Table 1.) According to the measurements before the supervised training (from the first and the last four weeks average results), the RHR lowered 67 bpm (from 76 bpm), systolic blood pressure (SBP) increased to 135 mmHg (from 126 mmHg), and diastolic blood pressure (DBP) decreased to 67 mmHg (from 72 mmHg). However, these changes were observed, besides the fact that the medical doctor prescribed the cessation of the medication for prediabetes (Meforal) and for hyperlipidemia (Fenoswiss) and halved the dose of medication for high blood pressure (Coverex-AS Komb) on week 16.

**Figure 1:**
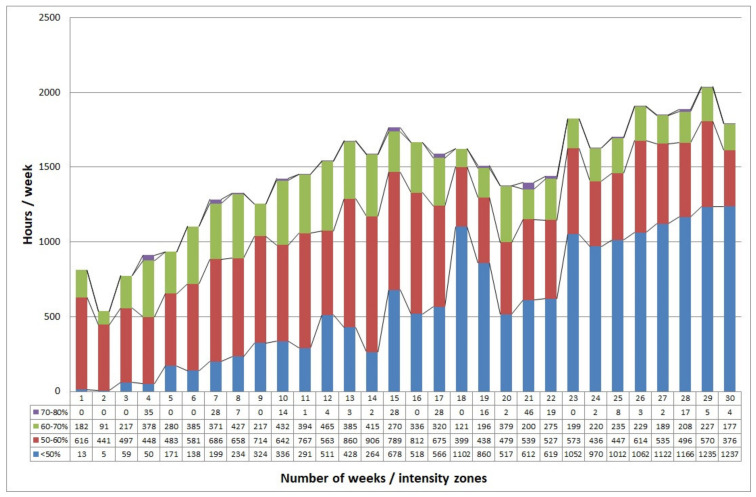
Training h/week spent in HR zones during the program. Maximum HR was determined from the 220-age formula, and intensity zones were calculated for the patient.

Fitness level increased to 7.7 MET and 26.9 mL/kg/min VO_2_max measured with the same protocol. Remarkable improvement in PA was monitored due to the data of the watch. Daily steps increased from 9133 steps to 16,087 steps (average of the first and last week), while time spent training increased from 13.5 h to 33.95 h a week of which that spent in moderate intensity increased drastically from 182 min to 446 min, then fluctuated (Figure 1). There was a significant positive correlation between the rate of weight loss and time spent in moderate-vigorous intensity (r = 0.52 *p* < 0.001). Besides this amount of training, there were no difficulties reaching the daily PA 100% at Level 3 (11 h 19 min low-intensity, 3 h 24 min medium-intensity, 1 h 29 min high-intensity physical activity, or these in combination) of the smartwatch.

## 4. Discussion

Lifestyle medicine can provide life-long improvement in health with the approach to change the attitude of obese patients [18]. Regular PA has a major part in it, but current research leads to several directions in the case of exercise therapy of patients with chronic diseases. They demonstrate that even 10–59 min of low-intensity exercise per week improves health [7], but for better results, it goes beyond 300 min of aerobic exercise [5]. This is a very broad range and to add the interindividual variability in response to the exercise [12], the details of the therapy—such as intensity, type of exercise, duration, or sets/repetitions—must be discussed and supervised by exercise professionals who are able to prescribe the correct “dose”, not just the beginning but through the therapy. Naturally, the other members of the lifestyle medicine team—medical doctors and nutritionists—are important for better results, as this case study also showed.

Activity and HR monitors are essential tools to measure PA objectively in order to individualize the therapy and for better understanding the dose-response effect of the exercise treatment [19,20]. Using them continuously with telemedicine solutions makes the non-supervised training intrinsically supervised, and it can increase the safeness and effectiveness of the exercise treatment [21].

After the 30-week program, the patient is still in obesity class II, with BMI 35.8 kg/m^2^, but achieved a significant decrease in cardio-metabolic risk factors. Other studies demonstrated that even a 10 kg weight reduction leads to the decrease in SBP 5 to 20 mmHg, risk of liver-related mortality [22], HbA1C and CVD risk factors [23], and further weight loss has greater improvement. However, cholesterol and triglyceride levels can be elevated after improvement in cases of major weight loss [24].

## 5. Conclusions

Although the exercise therapy of this patient is an extreme example with the 33.95 h per week training at the end of the program, the weight loss, improvement in blood test results, and decrease in medication can show the effects of complex lifestyle medicine treatment with supervised and monitored exercise therapy. During the exercise therapy, personalized training and safeness were the most important factors, despite performance increasing. The case demonstrates that the physiological values and quality of life of elderly patients with ABCD can also be significantly improved with the appropriate use of lifestyle medicine treatment. Although the role of regular exercise in health is well known, it is rarely used in clinical practice. The reasons can be that general PA recommendations show only minor results in health in the absence of individual exercise prescriptions. Furthermore, exercise professionals are not regular members of the medical team due to the lack of cooperation. However, this case study shows that the professional way of using exercise therapy is by means of longitudinal individualization and continuous monitoring.

However, there are some aspects that should be followed for the professional exercise therapy of ABCD patients. After the proper medical examinations, it should be started with submaximal fitness testing, led by an exercise professional. Due to its results, actual conditions, and medications, the safe target heart rate zone should be prescribed for the training. Additionally, depending on the fitness level and condition of the patient, the frequency of the training should start from 2 to 4 occasions a week. At least one training session a week should be supervised personally, but the others must be controlled by a heart rate monitor, the results of which can be shared with the exercise professional due to the safety and efficiency of the training. Intensity or duration should be increased by degrees, depending on the development of the patient—it must be individualized every week. The main goal of exercise therapy—besides its effects for health—is to teach the patient how to engage in regular training on their own in safe conditions.

In order to make a more precise, detailed exercise therapy recommendation for ABCD patients, more well-documented case studies are needed. They should include medical reports, medications, blood test results, and nutritional and exercise diaries to formulate the dose-response effect of lifestyle medicine interventions.

## Figures and Tables

**Table 1:** Results of blood test during the program.

	Week 0	Week 12	Week 31
Blood glucose (mmol/L)	6.5	5.5	6
Hemoglobin A1c (HbA1c) (%)	6.2	5.8	5.9
Cholesterol (mmol/L)	6.2	5.9	6.6
Triglyceride (mmol/L)	2.65	1.56	1.68
Glutamic oxaloacetic transaminase (GOT) (U/L)	27	21	17
Glutamic pyruvic transaminase (GPT) (U/L)	45	29	17
Gamma-glutamyl transferase (GGT) (U/L)	50	32	26

## Data Availability

All data generated and analyzed during this study are included in this manuscript.
